# Cross-phenotype integration of eQTL-based Mendelian randomization and transcriptomics prioritizes *BMP2K* in IgA vasculitis with nephritis

**DOI:** 10.3389/fimmu.2026.1757142

**Published:** 2026-09-18

**Authors:** Su Peng, Chuang Xie, Chuang Qian, Ping Yuan, Shiyou Guan, Xiangmei Zhang, Yan Chen, Pei Huang, Zuochen Du

**Affiliations:** 1 Department of Pediatrics, Affiliated Hospital of Zunyi Medical University, Zunyi, China; 2 Guizhou Children’s Hospital, Zunyi, China

**Keywords:** bmp2k, candidate-gene prioritization, IgA vasculitis with nephritis, Mendelian randomization, ruboxistaurin, transcriptomics

## Abstract

**Objective:**

To prioritize candidate genes and compounds potentially associated with IgA vasculitis with nephritis (IgAVN) by integrating genetic and transcriptomic evidence across IgAVN, IgA vasculitis (IgAV), and IgA nephropathy (IgAN), followed by preliminary cellular and pharmacological validation.

**Methods:**

Expression quantitative trait locus (eQTL)-based two-sample Mendelian randomization (MR) was performed using FinnGen summary statistics for IgAVN, IgAV, and IgAN. MR-prioritized genes were integrated with differentially expressed genes from the IgAVN transcriptomic dataset GSE102114. Fisher’s method was used to combine convergent evidence, and exploratory immune cell-specific single-cell eQTL-based MR was conducted to identify potential cell-type-relevant signals. Candidate compounds were screened using drug-signature analysis and evaluated by molecular docking. *BMP2K* mRNA expression was measured by RT-qPCR in peripheral blood mononuclear cells (PBMCs) from children with IgAVN (*n* = 13), children with IgAV (*n* = 10), and healthy controls (*n* = 13). Functional and pharmacological effects were evaluated in lipopolysaccharide (LPS)-stimulated THP-1 cells using siRNA-mediated knockdown, CCK-8, RT-qPCR, Western blotting, and ELISA.

**Results:**

MR analysis identified 124 genes with nominally significant risk-increasing associations with IgAVN, 132 with IgAV, and 119 with IgAN. Integration with IgAVN transcriptomic data prioritized four candidate genes: *BMP2K*, *GYPB*, *IGFBP7*, and *MISP3*. Among these, *BMP2K* was the only gene supported across all three IgA-related phenotypes and the IgAVN transcriptomic dataset. *BMP2K* mRNA expression was significantly higher in PBMCs from children with IgAVN than in healthy controls. Exploratory single-cell eQTL-based MR suggested a nominal monocyte-related association between genetically predicted *BMP2K* expression and IgAVN risk. In THP-1 cells, *BMP2K* knockdown reduced LPS-induced secretion of IL-6, IL-8, and TNF-α. Drug-signature analysis and molecular docking nominated ruboxistaurin as a candidate compound. Ruboxistaurin attenuated LPS-induced inflammatory cytokine expression and secretion and was associated with a statistically significant reduction in BMP2K expression.

**Conclusion:**

Integrative genetic, transcriptomic, and preliminary experimental evidence prioritized *BMP2K* as a candidate gene for IgAVN. Ruboxistaurin showed anti-inflammatory activity and was associated with lower BMP2K protein abundance *in vitro*; however, direct target engagement and BMP2K-specific activity were not established. Larger independent genetic datasets, colocalization analyses, renal tissue validation, and disease-relevant mechanistic models are required.

## Introduction

IgA vasculitis with nephritis (IgAVN) represents a clinically significant organ manifestation of IgA vasculitis (IgAV), a small-vessel vasculitis driven by immune complex deposition (1). Renal involvement commonly manifests as microscopic and/or gross hematuria, often accompanied by varying degrees of proteinuria, and severe or persistent cases may progress to chronic kidney disease or kidney failure (2). Although IgAV predominantly affects children, adults with IgAVN generally have a higher risk of unfavorable renal outcomes, partly because chronic histopathological lesions are more frequent and treatment responses may be less favorable (3). Histologically, IgAVN is characterized by mesangial IgA-dominant immune deposits, mesangial hypercellularity, and variable proliferative or chronic lesions (4).

The renal pathological and immunological features of IgAVN closely resemble those of IgA nephropathy (IgAN), suggesting partially shared mechanisms involving mucosal immune dysregulation, aberrant IgA1 O-glycosylation, formation of nephritogenic immune complexes, and complement activation (4). Increased circulating galactose-deficient IgA1 (Gd-IgA1) may act as an autoantigen, promote the generation of anti-glycan antibodies, and facilitate the formation and renal deposition of pathogenic immune complexes (5). Complement activation and inflammatory kidney injury have also been linked to glomerular complement deposition, endothelial activation, the fractalkine–CX3CR1 axis, and monocyte/macrophage recruitment (6). These observations suggest that systemic IgA abnormalities and local innate immune responses may jointly contribute to renal injury.

Genetic studies further support overlapping but incompletely defined susceptibility across IgA-mediated diseases. Common variation in *C1GALT1* and *C1GALT1C1* influences circulating Gd-IgA1 levels (7, 8), whereas *TNFSF13* and HLA class II loci have been associated with IgA-related traits, IgAN, or IgAV susceptibility (9, 10). However, most genetic and mechanistic evidence has been derived from IgAN or IgAV rather than IgAVN. IgAVN-specific genome-wide association study (GWAS) datasets remain small, and the molecular determinants that distinguish IgAVN from IgAN or IgAV without nephritis remain poorly defined. Consequently, clinically useful molecular biomarkers and mechanism-based therapeutic targets for IgAVN are still lacking, and treatment remains largely empirical (11).

Integrating genetically predicted gene expression with disease-associated transcriptomic changes may provide a practical strategy for prioritizing candidates in diseases for which large GWAS datasets are not yet available. Mendelian randomization leverages genetic variants employed as instrumental variables to evaluate associations between genetically predicted exposures and disease outcomes, thereby reducing the potential confounding and reverse causation bias (12). Nevertheless, its validity and statistical power depend on instrument strength and the size and quality of the outcome dataset. Given the limited number of IgAVN cases in currently available GWAS datasets, MR was used here primarily as a candidate-prioritization approach rather than as definitive evidence of causality.

Accordingly, we developed a cross-phenotype framework integrating eQTL-based MR analyses of IgAVN, IgAV, and IgAN with IgAVN transcriptomic data. Convergent candidates were further evaluated using immune cell-specific single-cell eQTL (sceQTL)-based MR, compound-signature prediction, molecular docking, and preliminary validation in patient-derived PBMCs and LPS-stimulated THP-1 cells. The study aimed to prioritize biologically plausible genes and candidate compounds for subsequent genetic replication, renal tissue validation, and mechanistic investigation rather than to establish definitive causal targets or clinically actionable therapies.

## Materials and methods

### Study design and data resources

This study used a multistage, hypothesis-generating framework integrating eQTL-based MR, transcriptomic analysis, cross-phenotype evidence integration, immune cell type-resolved eQTL-informed MR, candidate-compound prediction, molecular docking, and preliminary experimental validation. The overall workflow is shown in **Figure 1**. The initial analysis included 50,044 eQTL-derived gene-expression exposure units from individuals of European ancestry obtained from the IEU OpenGWAS project. GWAS summary statistics for IgAV, IgAVN, and IgAN were derived from the FinnGen study (13). The datasets included 939 IgAV cases and 405,762 controls, 101 IgAVN cases and 412,080 controls, and 653 IgAN cases and 411,528 controls, respectively. Transcriptomic data were sourced from the Gene Expression Omnibus (GEO), accession GSE102114 (14), which comprises peripheral-blood RNA-sequencing samples from six children with IgAVN and four healthy controls.

**Figure 1:**
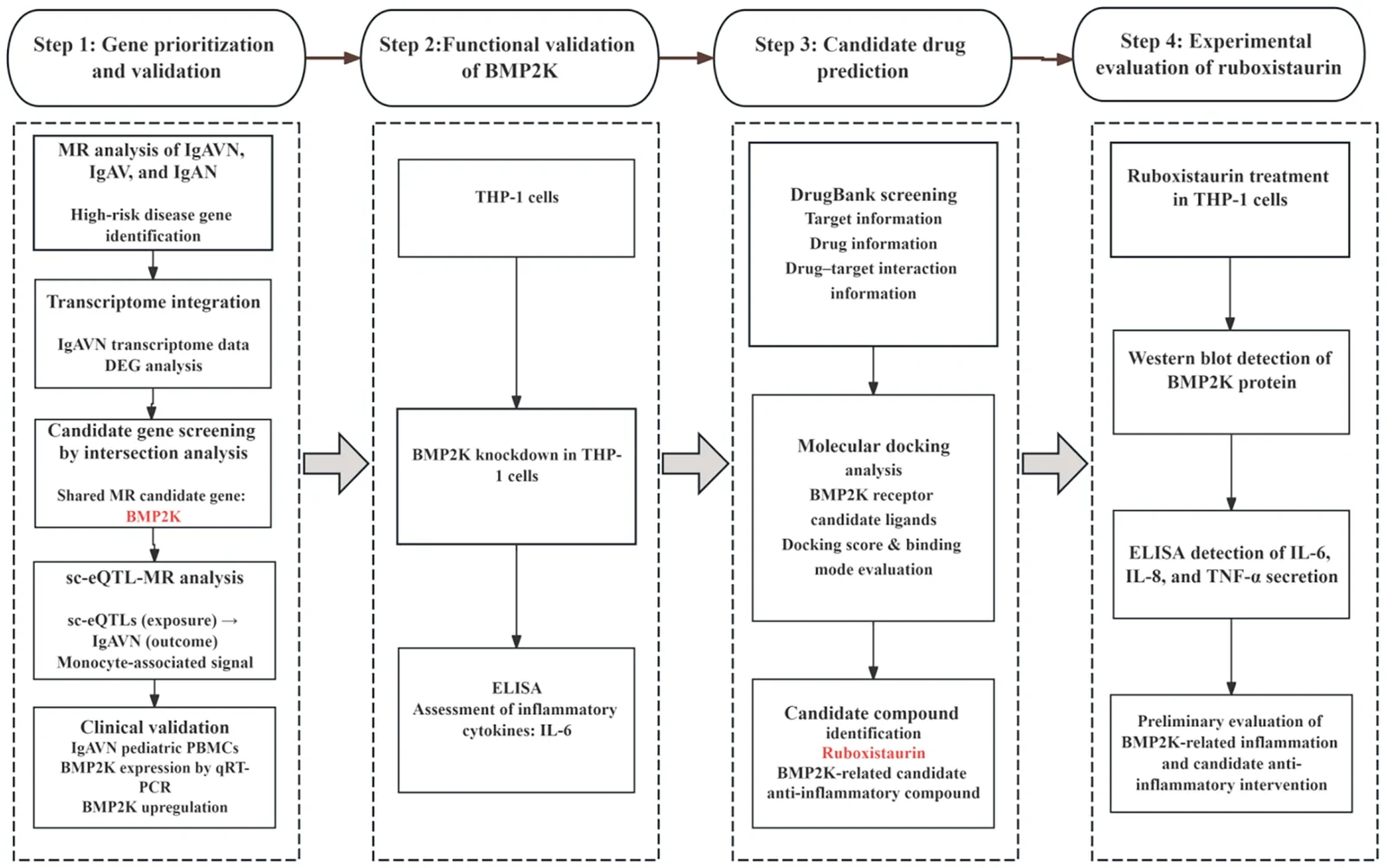
Overview of the study workflow. The study integrated eQTL-based MR, IgAVN transcriptomic analysis, overlap analysis of MR-prioritized genes and transcriptomic DEGs, Fisher’s method for evidence combination, immune cell-specific sceQTL-based MR, compound prediction, molecular docking, and preliminary RT-qPCR and cell-based validation.

### Instrumental variable selection

For bulk eQTL-based MR analysis, gene expression-associated variants meeting the genome-wide significance criterion (*P* < 5 × 10^-8^) were LD-clumped with linkage disequilibrium pruning performed at *r²* < 0.001 within a 10,000-kb window. Because fewer genome-wide significant variants were available for sceQTL analyses, sceQTL instruments were identified using a significance threshold of *P* < 1 × 10^-5^, followed by LD clumping with an *r²* cutoff of 0.1 across a 5,000-kb genomic window. Instrument strength was evaluated based on F-statistics, to reduce potential weak-instrument bias, variants with F-statistics of 10 or greater were retained, and the exposure and outcome summary statistics were harmonized with respect to the effect alleles; ambiguous palindromic variants were excluded when strand orientation could not be resolved.

### Two-sample Mendelian randomization analysis

Two-sample MR was performed using genetically predicted gene expression (15) as the exposure and IgAV, IgAVN, or IgAN as the outcome. The inverse-variance weighted method was used when at least two independent instrumental variables were available, whereas the Wald ratio was used for single-variant instruments. Effect estimates from the MR analyses were quantified using odds ratios (ORs) and their corresponding 95% confidence intervals (CIs). Genes with nominally significant risk-increasing associations (*P* < 0.05 and OR > 1) were retained as MR-prioritized candidates. Protective associations were outside the predefined scope of the present analysis. MR findings were used for candidate prioritization rather than as definitive evidence of causality.

### Transcriptomic analysis and identification of candidate genes

Differential expression analysis of the GSE102114 dataset was conducted in R using the DESeq2 package (16). The Benjamini–Hochberg procedure was applied to adjust raw P values for multiple comparisons. Differentially expressed genes (DEGs) were screened using the criteria of adjusted P < 0.05 and |log_2_ fold change| > 2. Four gene sets were compared: MR-prioritized genes associated with IgAVN, IgAV, and IgAN, and DEGs identified in the IgAVN transcriptomic dataset. Overlap among these gene sets was visualized using a Venn diagram, and genes supported by both MR and transcriptomic evidence were retained as cross-phenotype candidates.

### Integrated evidence assessment

Fisher’s method was used to combine the available MR and transcriptomic evidence for each cross-phenotype candidate (17). For a gene with *k* available tests, the combined statistic was calculated as χ² = −2∑_i=1_^k^ ln(*p*_i_), where p_i_ denotes the *P* value from the ith MR or transcriptomic analysis. Under the assumption of independence, the statistic is asymptotically distributed as χ² with 2k degrees of freedom. Combined *P* values were adjusted by Bonferroni correction for the four candidate genes. Because the phenotypes and component tests were not necessarily independent, the combined *P* values were used to rank convergent evidence rather than as formal evidence of pleiotropy or causality.

### Single-cell eQTL-based MR analysis

Human immune cell-specific eQTL data were obtained from scQTLbase (18). SceQTL instruments for *GYPB*, *BMP2K*, *IGFBP7*, and *MISP3* were selected according to statistical significance, LD independence, and instrument strength and were used in two-sample MR analyses with IgAVN as the outcome. These analyses were exploratory and were intended to identify potential immune cell-relevant regulatory signals rather than to establish cell-specific causality.

### Statistical power assessment

An approximate *post hoc* power analysis was performed in R using the pwr package under an assumed minor allele frequency of 0.20. For the IgAVN GWAS, the estimated power was 5.3% for an OR of 1.5, 8.1% for an OR of 2.0, and 15.7% for an OR of 2.5; power exceeded 80% only for very large effect sizes. These estimates were used descriptively and were not considered a formal MR-specific power analysis.

### Ethics approval and study participants

Ethical approval for the clinical component of this study was obtained from the Medical Research Ethics Committee of the Affiliated Hospital of Zunyi Medical University (approval no. ZMU 2024-1-232). Each participant gave written informed consent before participating in the study; when applicable, consent was obtained from their legally authorized representatives. Children with IgAV and IgAVN were enrolled according to the EULAR/PRINTO/PRES classification criteria (19). IgAVN was defined as IgAV accompanied by renal involvement, including hematuria, proteinuria, and/or biopsy-confirmed nephritis when biopsy results were available. Participants with another autoimmune disease, active allergic or chronic inflammatory disease, active infection, or exposure to systemic corticosteroids or immunosuppressive agents before blood collection were excluded. Age- and sex-matched healthy children were recruited as controls. The validation cohort included 10 children with IgAV, 13 with IgAVN, and 13 healthy controls.

### PBMC isolation

PBMCs were obtained from peripheral venous blood samples through centrifugation over a density gradient with lymphocyte separation medium (Cytiva Sweden AB, Sweden), following the manufacturer’s instructions, and subsequently used for RNA extraction and RT-qPCR analysis of *BMP2K* expression.

### Candidate-compound prediction and molecular docking

BMP2K-associated candidate compounds were identified through querying the Drug Signatures Database (DSigDB) (20). Gene–compound relationships were visualized as a network, and ruboxistaurin (LY-333531) was selected for molecular docking and experimental evaluation. The chemical structure of ruboxistaurin was retrieved from PubChem, and the tertiary structure of BMP2K was downloaded from the Protein Data Bank (PDB). Computational molecular docking was carried out using CB-Dock2; the predicted binding pose, docking score, and interacting residues were recorded. Docking was used to assess potential structural compatibility and was not interpreted as evidence of direct binding, intracellular target engagement, or BMP2K-specific inhibition.

### Cell culture

Human THP-1 monocytic cells were maintained in RPMI 1640 medium containing 10% heat-inactivated fetal bovine serum and cultured at 37 °C under humidified conditions containing 5% CO_2_ (21).

### siRNA-mediated *BMP2K* knockdown

Commercial human *BMP2K*-targeting siRNA oligonucleotide duplexes (HY-RS01537; MedChemExpress, Shanghai, China) were used to silence *BMP2K*, and a scrambled siRNA served as the negative control. THP-1 cells were first seeded into six-well plates and then subjected to transfection in reduced-serum medium following the manufacturer’s protocol. At 36 h after transfection, cells were stimulated with LPS (1.5 μg/mL) for 24 h. Cells were collected for Western blotting, and culture supernatants were subsequently harvested for ELISA quantification of IL-8, IL-6, and TNF-α levels. Experiments were independently repeated three times.

### Ruboxistaurin treatment

Ruboxistaurin (LY-333531; MedChemExpress, Shanghai, China) was used at a final concentration of 1 μM (22). THP-1 cells were pretreated with ruboxistaurin for 1 h, followed by stimulation with LPS (1.5 μg/mL) for 24 h. Cells were then collected for Western blotting and RT-qPCR analyses, and culture supernatants were collected for ELISA. To assess potential concentration-related effects on cellular metabolic activity under the same experimental conditions, THP-1 cells were pretreated with ruboxistaurin at 0, 0.5, 1, 2, 4, or 8 μM for 1 h, followed by LPS stimulation (1.5 μg/mL) for 24 h. Cell viability was assessed using the CCK-8 assay. All experiments were independently repeated three times.

### RNA extraction and RT-qPCR

Total RNA was isolated from PBMCs and THP-1 cells with TRIzol reagent (BioTeke, China), and complementary DNA was synthesized according to the PrimeScript RT Reagent Kit (Takara Bio, USA). RT-qPCR was performed with a CFX96 Real-Time PCR Detection System (Bio-Rad, USA). Relative mRNA levels were quantified using the comparative Ct method (2^-^ΔΔCt), with GAPDH serving as the internal reference gene. The primer sequences employed in this study are provided below:*GAPDH*, forward 5′-AATGGGCAGCCGTTAGGAAA-3′ and reverse 5′-GCCCAATACGACCAAATCAGAG-3′; *BMP2K*, forward 5′-CCATCACCACCAAGGCTGAT-3′ and reverse 5′-TCTGGGATGGTGAAGTTGCC-3′; *IL6*, forward 5′-ACTCACCTCTTCAGAACGAATTG-3′ and reverse 5′-CCATCTTTGGAAGGTTCAGGTTG-3′; *CXCL8*, forward 5′-ACTGAGAGTGATTGAGAGTGGAGAC-3′ and reverse 5′-AACCCTCTGCACCCAGTTTTC-3′; and *TNF*, forward 5′-CCTCTCTCTAATCAGCCCTCTG-3′ and reverse 5′-GAGGACCTGGGAGTAGATGAG-3′.

### Enzyme-linked immunosorbent assay

The collected culture supernatants were clarified by centrifugation at 500 × g for 5 min at 4 °C to remove residual cells and cellular debris. IL-6, TNF-α, andIL-8 concentrations were measured using commercial ELISA kits (4A Biotech, China; catalog nos. CHE009, CHE0011, and CHE0019, respectively) after conducting the assay in accordance with the manufacturer’s instructions, absorbance was measured at 450 nm using a BioTek Synergy H1 microplate reader. Samples were measured in technical triplicate, and coefficients of variation below 10% were considered acceptable.

### Western blotting

Total cellular protein lysates were solubilized from THP-1 cells using radioimmunoprecipitation assay (RIPA) lysis buffer (G2002; Servicebio, China) containing protease and phosphatase inhibitor cocktails(G2006 and G2007, respectively; Servicebio, China). Equivalent amounts of total protein were resolved by electrophoresis on 10% SDS-PAGE gels and then transferred onto PVDF membranes. To minimize nonspecific antibody binding, the membranes were incubated with 5% non-fat milk and subsequently probed overnight at 4 °C with an anti-*BMP2K* primary antibody (32853-1-AP; Proteintech, China). The membranes were then treated with an HRP-linked goat anti-rabbit IgG secondary antibody (RGAR001; Proteintech, China).Protein signals were detected by enhanced chemiluminescence imaging, and β-actin was used as the internal loading reference. Representative blot images from three independently repeated experiments are presented. Band intensities from three independent experiments were quantified using ImageJ and normalized to the corresponding β-actin loading control.

### Statistical analysis

R software (v4.3.3) and GraphPad Prism (v10.0) were used for statistical processing and analysis. Mendelian randomization analyses were implemented using the TwoSampleMR package, Differential expression profiling was carried out using DESeq2, and genes meeting the predefined significance criteria were retained for subsequent analyses. MR estimates are expressed as odds ratios (ORs),with corresponding 95% confidence intervals (CIs). Significantly altered genes were identified based on an |log_2_FC| > 2 and BH-adjusted *P* < 0.05, the combined *P* values obtained using Fisher’s method were subsequently corrected with the Bonferroni procedure. Densitometric quantification of immunoblot bands was performed using ImageJ, normalized to the corresponding loading control. Data are reported as the mean ± SD from three independent biological replicates unless noted otherwise. Comparisons between two groups were performed using a two-tailed Student’s t-test or, when appropriate, the Mann–Whitney U test. For comparisons across three or more groups, one-way ANOVA was used for data satisfying parametric assumptions, whereas the Kruskal–Wallis test was applied to nonparametric data; *post hoc* multiple-comparison analyses were then performed as appropriate. All hypothesis tests were performed as two-sided tests, and results were considered significant when *P* < 0.05.

## Results

### Integrative prioritization and preliminary validation of genes associated with IgAVN

The overall analytical and experimental workflow is summarized in **Figure 1**. Among the 50,044 eQTL-derived exposure units evaluated, two-sample MR identified 124 genes with nominally significant risk-increasing associations with IgAVN, 132 with IgAV, and 119 with IgAN. Differential expression analysis of GSE102114 identified 2,993 DEGs, including 2,185 upregulated and 808 downregulated genes.

The MR-prioritized gene sets for IgAVN, IgAV, and IgAN were integrated with the IgAVN DEGs. *GYPB*, *BMP2K*, and *IGFBP7* were shared between IgAVN and IgAV, whereas *MISP3* and *BMP2K* were shared between IgAVN and IgAN. *BMP2K* was the only gene supported by MR evidence across all three IgA-related phenotypes and by differential expression in the IgAVN transcriptomic dataset (**Figure 2A**). MR effect estimates for the overlapping candidates are shown in **Figure 2B**.

**Figure 2:**
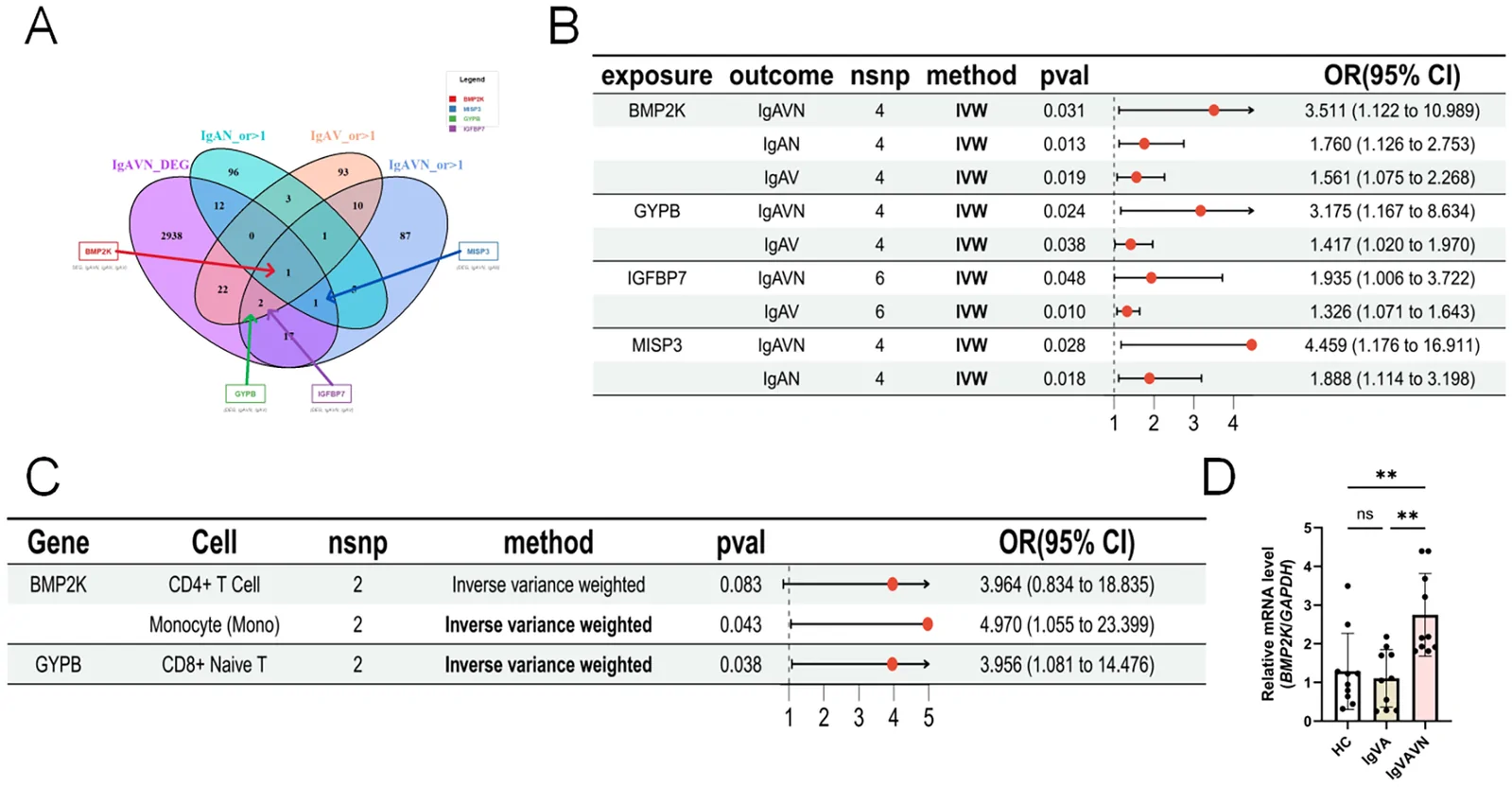
Integrative prioritization and preliminary validation of genes associated with IgAVN. **(A)** Overlap among MR-prioritized genes associated with IgAVN, IgAV, and IgAN and DEGs identified in the IgAVN transcriptomic dataset. **(B)** MR association estimates for the overlapping candidates across the three IgA-related phenotypes. **(C)** Exploratory immune cell-specific sceQTL-based MR showing immune cell-relevant signals for *BMP2K* and *GYPB*. **(D)** RT-qPCR analysis of *BMP2K* mRNA expression in PBMCs from healthy controls (HCs; *n* = 13), patients with IgAV (*n* = 10), and patients with IgAVN (*n* = 13). Data are presented as mean ± SD. ns, not significant; **P < 0.01.

Fisher’s method was used to combine the available MR *P* values with the IgAVN differential-expression *P* value for each candidate (23). The combined *P* values were 3.40 × 10^-9^ for *GYPB*, 3.93 × 10^-7^ for *BMP2K*, 4.35 × 10^-4^ for *IGFBP7*, and 1.43 × 10^-^³ for *MISP3*. All four candidates remained significant after Bonferroni correction, with adjusted *P* values of 1.36 × 10^-8^, 1.57 × 10^-6^, 1.74 × 10^-^³, and 5.71 × 10^-^³, respectively (**Table 1**). Because the phenotypes and component tests may not be fully independent, the combined *P* values were used to rank convergent evidence rather than to establish biological pleiotropy or causality.

**Table 1:** Fisher combined *P* values used to rank integrated candidate-gene evidence.

Gene	IgAVN P	IgAV P	IgAN P	DEG P	Raw P	Adjusted P	Bonferroni significant
*BMP2K*	0.031	0.019	0.013	2.38 × 10^-5^	3.93 × 10^-7^	1.57 × 10^-6^	Yes
*MISP3*	0.027	—	0.018	4.19 × 10^-^²	1.43 × 10^-^³	5.71 × 10^-^³	Yes
*GYPB*	0.023	0.037	—	1.15 × 10^-8^	3.40 × 10^-9^	1.36 × 10^-8^	Yes
*IGFBP7*	0.047	0.009	—	1.17 × 10^-^²	4.35 × 10^-4^	1.74 × 10^-^³	Yes

Fisher’s method combined the available MR *P* values for each candidate across the relevant IgA-related phenotypes with the differential-expression *P* value from GSE102114. Raw *P* is the unadjusted combined *P* value, and Adjusted *P* is the Bonferroni-corrected value for four tested genes. Because the component tests may be correlated, these values were used for evidence ranking and do not establish pleiotropy or causality.

Exploratory MR analyses based on immune cell-specific sceQTL data were then performed. Genetically predicted higher *BMP2K* expression in monocytes was nominally associated with increased IgAVN risk (OR = 4.97, 95% CI = 1.06–23.40, *P* = 0.043), although the estimate was based on only two instrumental variables. In CD4^+^ T cells, genetically predicted *BMP2K* expression showed a positive but nonsignificant association with IgAVN risk (OR = 3.96, 95% CI = 0.83–18.84, *P* = 0.083). Genetically predicted higher *GYPB* expression in naïve CD8^+^ T cells was also nominally associated with increased IgAVN risk (OR = 3.96, 95% CI = 1.08–14.48, *P* = 0.038) (**Figure 2C**). Given the limited number of instruments, wide CIs, and relaxed instrument-selection threshold, these findings should be interpreted as exploratory cell-type-relevant signals rather than evidence of cell-specific causality.

*BMP2K* mRNA expression was then measured in PBMCs from children with IgAVN (*n* = 13), children with IgAV (*n* = 10), and healthy controls (*n* = 13). Expression was significantly higher in the IgAVN group than in healthy controls, whereas no significant difference was detected between the IgAV and healthy-control groups (**Figure 2D**). This directionally concordant result provided additional support for prioritizing *BMP2K* for subsequent functional investigation.

Collectively, *BMP2K* showed the most consistent support across cross-phenotype MR, IgAVN transcriptomics, Fisher evidence integration, exploratory immune cell-specific sceQTL analysis, and preliminary PBMC expression validation and was therefore selected for functional and pharmacological investigation.

### *BMP2K* knockdown suppresses LPS-induced release of IL-6, IL-8, and TNF-α by THP-1 cells

THP-1 cells were introduced with control siRNA or *BMP2K*-targeting siRNA and subsequently stimulated with LPS. Western blotting followed by densitometric quantification demonstrated significantly lower BMP2K protein abundance after *BMP2K*-targeting siRNA transfection than after control siRNA transfection (**Figures 3A, B**). ELISA analysis showed that *BMP2K* knockdown significantly reduced LPS-stimulated secretion of IL-8, IL-6, and TNF-α (**Figures 3C–E**). These findings suggest that BMP2K contributes to the LPS-induced inflammatory response in THP-1 cells, although the downstream signaling mechanism remains to be established.

**Figure 3:**
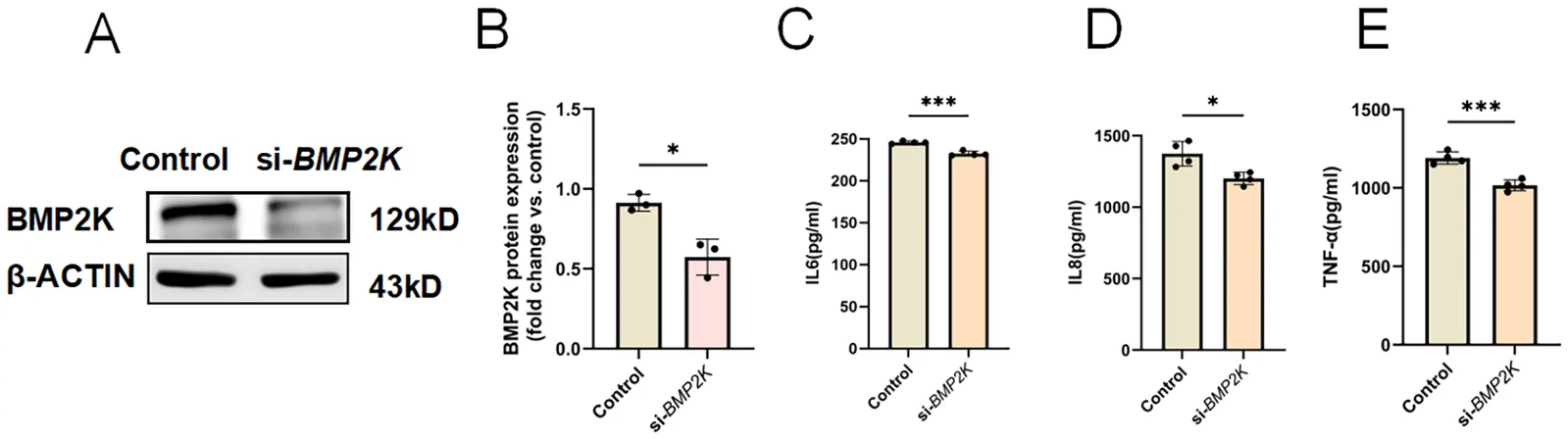
*BMP2K* knockdown suppresses LPS-induced IL-6, IL-8, and TNF-α secretion in THP-1 cells.**(A)** Representative Western blot analysis of BMP2K protein expression in THP-1 cells transfected with control siRNA or *BMP2K*-targeting siRNA followed by LPS stimulation. The immunoblots shown are representative of three independent experiments. **(B)** Densitometric quantification of BMP2K protein expression normalized to β-actin from three independent experiments. **(C–E)** Concentrations of IL-6, IL-8, and TNF-α in the culture supernatants of THP-1 cells transfected with control siRNA or BMP2K-targeting siRNA following LPS stimulation, as measured by ELISA. Data are presented as the mean ± SD from three independent experiments. *P < 0.05, **P < 0.01, ***P < 0.001.

### Compound prediction and molecular docking analysis

BMP2K was selected for candidate-compound screening on the basis of the integrative prioritization results. DSigDB identified a network of BMP2K-associated compounds, including GSK-461364, GW843682, LY-333531, GSK461364, AS601245, AG-013736, TG-100-115, XMD13-2, SP600125, and AR-A014418 (**Figure 4A**). LY-333531, also known as ruboxistaurin, was selected for molecular docking and experimental evaluation.

**Figure 4:**
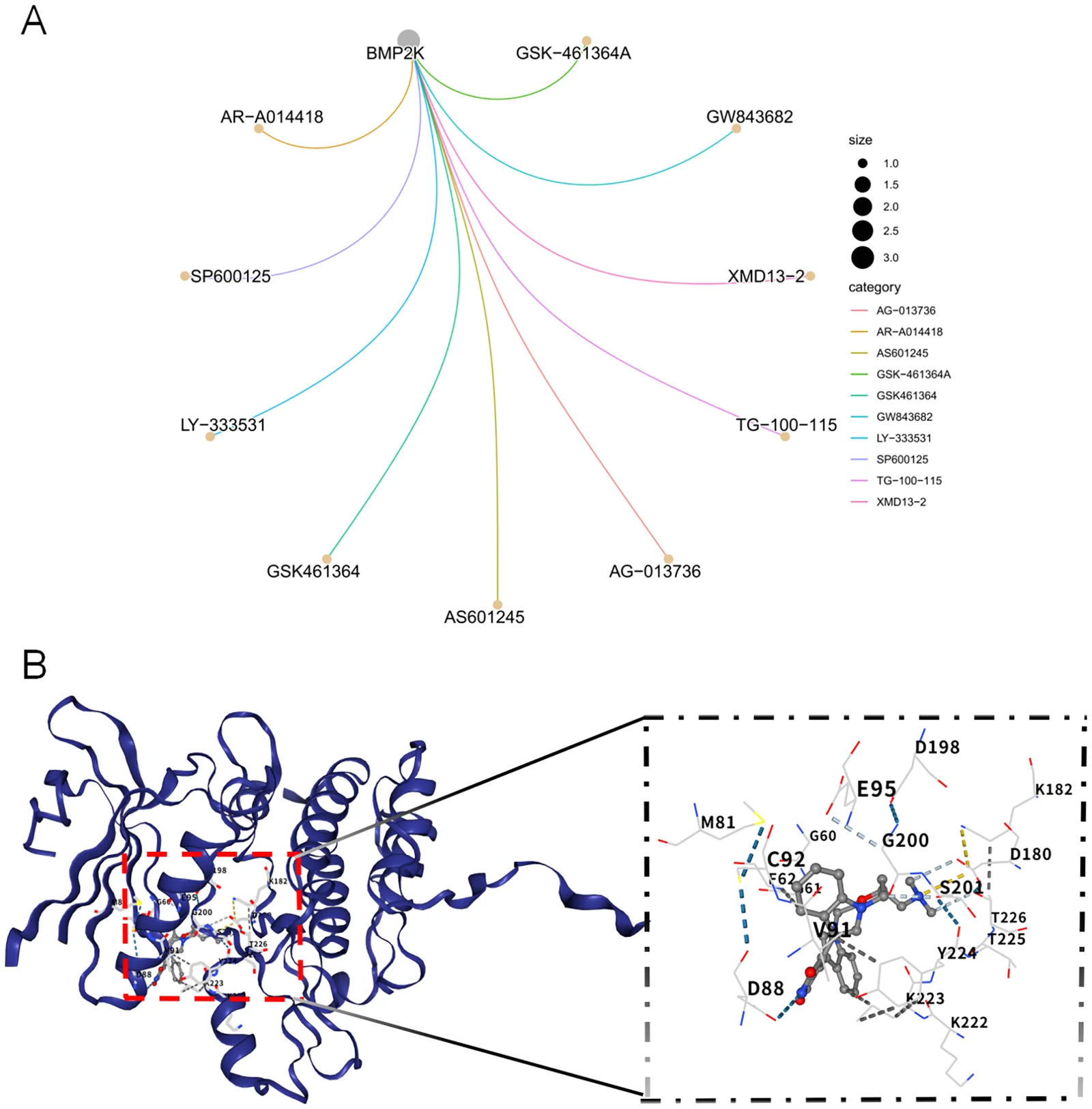
Compound prediction and molecular docking analysis. **(A)** Network of candidate compounds associated with BMP2K identified using DSigDB. **(B)** Predicted binding pose of ruboxistaurin (LY-333531) within a putative BMP2K binding pocket, together with an enlarged view of the predicted interacting residues. Molecular docking provides computational evidence of structural compatibility but does not establish direct target engagement or therapeutic efficacy.

Molecular docking predicted a favorable pose of ruboxistaurin within a putative BMP2K binding pocket, with a docking score of −9.1 kcal/mol and several predicted interacting residues (**Figure 4B**). This result supported experimental testing but did not demonstrate direct target engagement, BMP2K-specific inhibition, or therapeutic efficacy.

### Ruboxistaurin attenuates LPS-induced inflammatory responses and is associated with lower BMP2K protein abundance in THP-1 cells

To assess potential concentration-related nonspecific effects, THP-1 cells were treated with increasing concentrations of ruboxistaurin under the same experimental conditions used for the pharmacological studies. Ruboxistaurin treatment at 0.5 or 1 μM had no significant effect on cell viability, whereas treatment at 2, 4, and 8 μM significantly decreased cell viability (**Figure 5A**). Thus, 1 μM was selected as the highest concentration that did not significantly reduce CCK-8 absorbance. This concentration range is consistent with a previous study in BMMs, in which ruboxistaurin concentrations ≤1 μM did not affect CCK-8-based viability measurements, whereas concentrations ≥ 2 μM resulted in dose-dependent reductions in cell viability (22).

**Figure 5:**
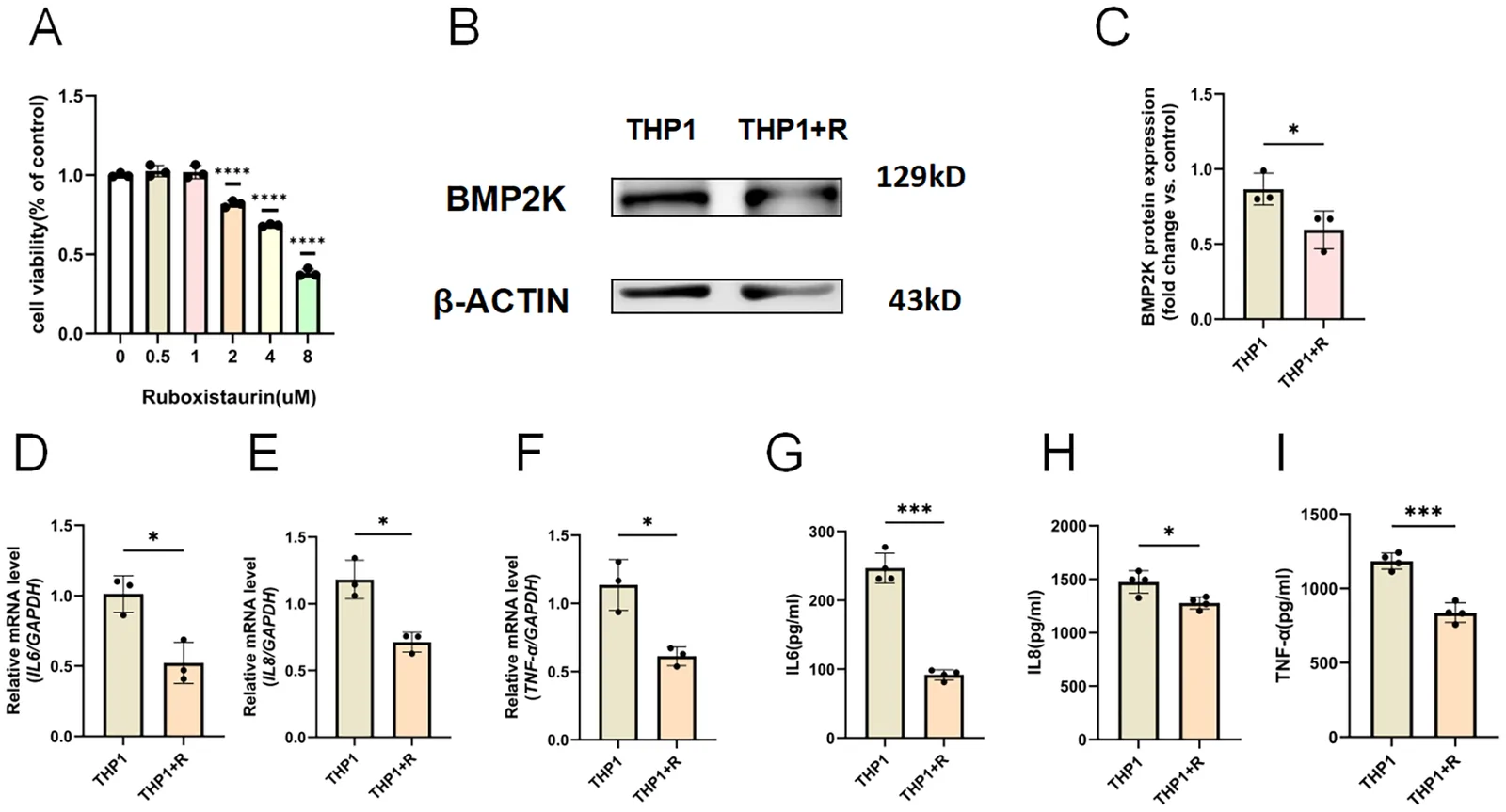
Ruboxistaurin attenuates LPS-induced inflammatory responses and is associated with lower BMP2K protein abundance in THP-1 cells. **(A)** Dose-response effect of ruboxistaurin on THP-1 cellular metabolic activity measured by CCK-8. **(B)** Representative Western blot analysis of BMP2K protein abundance in THP-1 cells pretreated with or without ruboxistaurin (R) and subsequently stimulated with LPS. The immunoblot shown is representative of three independent experiments. **(C)** Densitometric quantification of BMP2K protein expression normalized to β-actin from three independent experiments.**(D–F)** RT-qPCR analysis of *IL6*, *CXCL8*, and *TNF-α* mRNA expression in THP-1 cells pretreated with or without ruboxistaurin before LPS stimulation. **(G–I)** ELISA quantification of IL-6, IL-8, and TNF-α concentrations in culture supernatants from THP-1 cells pretreated with or without ruboxistaurin before LPS stimulation. Data are presented as mean ± SD from three independent experiments. ns, not significant; *P < 0.05, **P < 0.01, and ***P < 0.001.

THP-1 cells were pretreated with or without ruboxistaurin before LPS stimulation. Western blotting showed lower BMP2K protein abundance in ruboxistaurin-treated cells, and densitometric quantification confirmed a statistically significant reduction (**Figures 5B, C**). These findings indicate that ruboxistaurin treatment is associated with reduced BMP2K protein expression but do not establish a direct interaction between ruboxistaurin and BMP2K. RT-qPCR showed that ruboxistaurin altered the LPS-induced mRNA expression of *IL6*, *CXCL8*, and *TNF*, with significant reductions in all three transcripts (**Figures 5D–F**). ELISA demonstrated that ruboxistaurin significantly reduced IL-6, IL-8, and TNF-α concentrations in culture supernatants from LPS-stimulated THP-1 cells (**Figures 5G–I**).

Together, these data show that ruboxistaurin attenuates inflammatory cytokine responses elicited by LPS in THP-1 cells and is associated with a statistically significant reduction in BMP2K protein abundance by densitometric quantification. Because ruboxistaurin is an established protein kinase C-β inhibitor and direct BMP2K engagement was not tested, the findings do not establish BMP2K-specific or exclusively BMP2K-dependent activity.

## Discussion

This study integrated genetic, transcriptomic, cellular, and pharmacological evidence to prioritize candidates potentially relevant to IgAVN. Four genes—*GYPB*, *BMP2K*, *IGFBP7*, and *MISP3*—were supported by MR–transcriptomic overlap, and *BMP2K* was the only candidate supported across IgAVN, IgAV, IgAN, and the IgAVN transcriptomic dataset. Higher *BMP2K* mRNA expression in PBMCs from children with IgAVN and the attenuation of LPS-induced cytokine secretion after *BMP2K* knockdown provided preliminary experimental support. These findings justify further study of *BMP2K* but do not establish a causal, kidney-specific, or therapeutically actionable role.

The exploratory sceQTL-based MR analysis suggested monocytes as a potentially relevant cellular context. However, the association was nominal, based on only two instruments, and accompanied by a wide CI. It therefore cannot establish monocyte-specific causality. The biological context is nevertheless plausible because monocytes and macrophages contribute to endothelial activation, glomerular inflammation, immune-complex handling, and tissue injury in immune-mediated kidney disease (6, 24). The PBMC validation was consistent with the transcriptomic analysis, but the small cross-sectional cohort and absence of kidney tissue or longitudinal data preclude conclusions about intrarenal expression, diagnostic performance, or prognostic value.

BMP2K encodes BMP-2-inducible kinase, a member of the numb-associated kinase family implicated in kinase signaling, membrane trafficking, and endocytosis. Altered BMP2K expression has been reported in studies of asymmetric dimethylarginine-related endothelial dysfunction (25), obesity-associated adipose tissue (26), and diabetic nephropathy (27). These observations place BMP2K at a possible intersection of vascular stress, metabolism, and renal injury, but none directly implicates it in IgAVN. Its localization and function in renal endothelial cells, podocytes, mesangial cells, tubular epithelial cells, and infiltrating leukocytes remain unknown.

In THP-1 cells, *BMP2K* knockdown reduced LPS-Mediated Secretion of IL-6, TNF-α, and IL-8, suggesting that BMP2K may contribute to amplification of innate inflammatory responses in this model. These cytokines participate in leukocyte recruitment (28), endothelial activation (29), and tissue inflammation (30); however, the present data do not define the molecular pathway linking BMP2K to cytokine production. Rescue experiments, gain-of-function studies, kinase-inactive mutants, and pathway analyses are needed to determine whether the phenotype depends on BMP2K kinase activity.

The LPS model also limits disease specificity. LPS induces acute Toll-like receptor 4-mediated innate immune activation (31), whereas IgAVN involves Gd-IgA1-containing immune complexes, complement activation, endothelial injury, and renal inflammation (1). Future studies should therefore evaluate BMP2K in primary human monocytes and macrophages stimulated with IgA-containing immune complexes or patient-derived IgA and in co-culture systems incorporating kidney-resident cells.

Drug-signature analysis and molecular docking nominated ruboxistaurin (LY-333531) for experimental evaluation. Ruboxistaurin is a protein kinase C-β inhibitor that has been studied in diabetic microvascular disease and experimental kidney injury (32, 33); PKC-β inhibition has been associated with reduced albuminuria and attenuation of profibrotic or inflammatory signaling (34). The docking score of −9.1 kcal/mol suggested structural compatibility with the modeled BMP2K pocket, but docking alone cannot demonstrate direct binding, intracellular target engagement, kinase inhibition, or pharmacological selectivity.

Ruboxistaurin attenuated LPS-induced inflammatory cytokine expression and secretion and was accompanied by a statistically significant reduction in BMP2K protein abundance by densitometric analysis. Despite this quantitative reduction, direct evidence of the interaction between ruboxistaurin and BMP2K remains lacking, as no direct binding assay, kinase assay, or target-engagement experiment was performed. Because PKC-β is an established target of ruboxistaurin (35), the anti-inflammatory effects may reflect canonical PKC-β inhibition or broader kinase-network modulation. Ruboxistaurin should therefore be considered a candidate compound associated with BMP2K modulation rather than a validated BMP2K inhibitor.

Several limitations warrant emphasis. The IgAVN GWAS included only 101 cases and had low estimated power for modest effects; Accordingly, the Mendelian randomization (MR) findings, especially estimates with odds ratios (ORs) below 3.0, should be viewed as hypothesis-prioritization signals rather than definitive causal evidence. Initial screening used nominal significance and carried forward only risk-increasing associations. Differences in ancestry, tissue source, sample size, and phenotype definition among the eQTL, GWAS, and transcriptomic datasets may introduce heterogeneity. The relaxed sceQTL instrument threshold, limited instrument numbers, and wide CIs further reduce the robustness of cell-type-specific estimates. Fisher’s method combined potentially correlated tests and was therefore used for evidence ranking rather than formal meta-analysis. Colocalization was not performed, so it remains uncertain whether the eQTL and disease signals share the same causal variant.

The experimental evidence is also preliminary. The transcriptomic dataset and PBMC cohort were small and lacked independent replication, longitudinal sampling, and renal tissue confirmation. THP-1 cells do not fully reproduce primary monocytes, tissue macrophages, or the renal immune microenvironment, and LPS does not model IgA immune complex-mediated nephritis. No rescue experiment, structurally matched inactive compound, independent BMP2K-directed compound, or kidney-relevant functional endpoint was included. These limitations restrict extrapolation of the *in vitro* findings to IgAVN pathogenesis or treatment.

Future studies should replicate the genetic signals in larger and ancestrally diverse cohorts and incorporate colocalization and fine-mapping. Kidney biopsy, urinary-cell, single-cell, and spatial transcriptomic studies could clarify the cellular source and clinical relevance of BMP2K. Mechanistic work should extend to primary monocytes/macrophages and kidney-resident cells under IgA immune complex-related conditions. For ruboxistaurin, direct BMP2K binding and kinase-inhibition assays, target-engagement and selectivity studies, lower-concentration dose-response experiments with orthogonal viability assessment, and *in vivo* efficacy studies are required before therapeutic implications can be considered.

## Conclusion

*BMP2K* emerged as a cross-phenotype candidate supported by MR, transcriptomic, exploratory sceQTL, and preliminary experimental evidence. *BMP2K* knockdown attenuated LPS-induced secretion of IL-6, IL-8, and TNF-α in THP-1 cells. Ruboxistaurin showed anti-inflammatory activity and was associated with a statistically significant reduction in BMP2K protein abundance by densitometric analysis, but its direct relationship with BMP2K remains unresolved. Independent genetic replication, renal tissue validation, mechanistic studies, and direct pharmacological target assessment are required before causal or therapeutic conclusions can be established.

## Data Availability

The original contributions presented in the study are included in the article/**Supplementary Material**. Further inquiries can be directed to the corresponding authors.

## Supplementary material

Supplementary Figure 1 Uncropped/full-length Western blot source images. Full-length BMP2K and β-actin immunoblots from the BMP2K-knockdown and ruboxistaurin-treatment experiments are shown.
